# Mining TCGA and GEO databases for the prediction of poor prognosis in lung adenocarcinoma based on up-regulated expression of *TNS4*

**DOI:** 10.1097/MD.0000000000031120

**Published:** 2022-10-21

**Authors:** Feng Liu, Xinliang Gao, Wei Liu, Wujun Xue

**Affiliations:** a Department of Kidney Transplantation, Nephropathy Hospital, the First Affiliated Hospital of Xi’an Jiaotong University, Xi’an, PR China; b Department of Thoracic Surgery, Wuxi People’s Hospital Affiliated to Nanjing Medical University, Wuxi, PR China; c Department of Thoracic Surgery, The First Hospital of Jilin University, Changchun, PR China.

**Keywords:** focal adhesion, lung adenocarcinoma, prognosis, *TNS4*

## Abstract

To investigate the clinical significance of Tensin4 (*TNS4*) in human cancers, particularly lung cancer, we mined the Cancer Genome Atlas database for lung adenocarcinoma (TCGA-LUAD) and the Gene Expression Omnibus database to predict poor prognosis based on the up-regulated expression of *TNS4* in LUAD. The correlation between the clinical pathologic features of patients and *TNS4* gene expression was analyzed using the Wilcoxon signed-rank test. Cox regression analysis was used to evaluate the association of clinicopathologic characteristics with the overall survival (OS) of cancer patients using TCGA data. The relationship between *TNS4* expression and cancer patient survival was evaluated with Kaplan–Meier survival curves and meta-analyses. GO and KEGG were also included in the data mining methods. The expression level of *TNS4* in LUAD tissue was higher than that in adjacent normal tissue (*P* < .001). According to the Kaplan–Meier survival curve, LUAD patients with high *TNS4* expression had worse prognosis than those with low *TNS4* expression (*P* < .001 for OS; *P* = .028 for progression-free survival). A positive correlation between *TNS4* expression and poor OS was found with both univariate and multivariate analyses. Increased *TNS4* expression in LUAD was closely correlated with a higher disease stage (*P* = .007), positive lymph nodes (*P* = .005), and larger tumor size (*P* = .002). Moreover, meta-analysis including seven independent datasets showed LUAD patients with higher *TNS4* had poorer OS (combined hazard ratio = 1.27, 95% confidence interval 1.16–1.39). In the high-*TNS4* population, regulation of the actin cytoskeleton, extracellular matrix receptor interactions, and focal adhesion were differentially enriched. Integrin *α*6*β*4 and laminin-5 genes were also associated with *TNS4*. *TNS4* expression may be a potential biomarker for predicting poor survival in LUAD. Moreover, the correlation between *TNS4* and integrin *α*6*β*4 may be attributed to the role of *TNS4* in LUAD.

## 1. Introduction

With a 2-year survival rate of 35% to 44% and a 5-year survival rate of 18%, lung cancer is one of the leading causes of cancer-related deaths in humans.^[1,2]^ In 2020, there were 228,820 new diagnoses and 135,720 cancer-related deaths from lung and bronchial cancer in the United States.^[3]^ In recent years, lung adenocarcinoma (LUAD) has become the most prevalent subtype of lung cancer.^[4]^ Many genes have been found to promote the proliferation or inhibit the death of lung cancer cells through various pathways, thereby participating in the occurrence and development of LUAD. Targeting the carcinogenic mechanisms mediated by these genes can provide new directions for LUAD treatment.

*TNS4* (tensin-4, also known as *Cten*) is a protein coding gene belonging to the tensin family. *TNS4* is involved in cell migration, cartilage development, and communication in the cytoskeleton. Studies on the action mechanisms of tensins revealed that the members of this family use their Src Homologous 2 domain to interact with phosphotyrosine-containing proteins. They also bind to *β*-integrin tails via their phosphotyrosine binding domains,^[5]^ thereby regulating various biological processes, including proliferation, apoptosis, differentiation, cell adhesion, migration, and invasion.^[6]^ Over-expression of *TNS4* has been detected in human cancers, such as gastric cancer, esophageal cancer, hepatocellular carcinoma, colorectal cancer, breast cancer primary melanoma, and pancreatic cancer^[7–14]^ but the protein is down-regulated in prostate cancer.^[15]^ Elevated expression of *TNS4* has also been also found in lung cancer.^[16]^ Elevated *TNS4* expression promotes the tumorigenesis and development of lung cancer via inducing and stabilizing epithelial-mesenchymal transition.^[17,18]^ It has been reported that high expression of *TNS4* affects the prognosis of LUAD.^[19,20]^ However, the correlation of *TNS4* expression with poor LUAD prognosis is still not fully understood due to a lack of comprehensive analysis using multiple datasets and studies on the potential mechanism underlying the role of *TNS4* in the prognosis of LUAD.

To this end, we aimed to evaluate the prognostic significance of *TNS4* expression in LUAD by using a bioinformatics approach to analyze the differential expression of *TNS4* mRNA using the Cancer Genome Atlas database for lung adenocarcinoma (TCGA-LUAD), with comparisons between LUAD tissues and normal tissues. In addition, to explore the overall prognostic significance of *TNS4* in LUAD, a comprehensive meta-analysis was performed using data from the Gene Expression Omnibus (GEO) database. To investigate the potential mechanism underlying the role of *TNS4* in the prognosis of LUAD, enrichment analysis was conducted using the gene ontology (GO) and Kyoto encyclopedia of genes and genomes (KEGG) pathways. Our study provides direct evidence for the up-regulation of *TNS4* expression as a prognostic biomarker for LUAD patients as well as a potential therapeutic target for LUAD.

## 2. Material and Methods

### 2.1. Data mining from public databases

In this study, the RNA data (HTSeq-FPKM) for TCGA-LUAD and related clinical information were mainly obtained from the University of California Santa Cruz Xena website. The LUAD mRNA microarray data and the corresponding clinical data were obtained from the GEO database. The inclusion criteria for these datasets were GEO series (GSE) with more than 100 LUAD cases and GSE with overall survival (OS) and transcriptional data. Lung cancer samples in the TCGA database were included for further survival analysis and to evaluate the clinical relationships in the high and low *TNS4* expression groups. Our research is based on open-source data and therefore does not require ethics committee approval for the study.

### 2.2. Meta-analysis

To evaluate the prognostic significance of *TNS4* in LUAD patients, meta-analysis was performed using both TCGA and GEO datasets. To evaluate the correlation of *TNS4* expression with the prognosis of LUAD patients, the hazard ratio (HR) and 95% confidence interval (CI) were calculated. To assess the heterogeneity across datasets, the Q test (I2 statistics) was performed. In the event of no obvious heterogeneity (I2 < 50%), a fixed-effects model was selected. Otherwise, a random-effects model was applied. The R software package “meta” was used for meta-analysis.

### 2.3. Enrichment analysis

In the TCGA-LUAD cohort, differentially expressed genes (DEGs) were identified by comparing the patients in the high-risk and low-risk groups using the “limma” package in R software. For the candidate genes, that is, DEGs with |log2 fold change| ≥1, GO enrichment analysis was performed using the “ClusterProfiler” package in R software, with a false discovery rate (FDR) < 0.05. Gene set enrichment analysis (GSEA) of the DEGs was also carried out for gene candidates that met the requirements (|log2 fold change| ≥ 1 and FDR < 0.05 in the KEGG pathway analysis). The correlation between *TNS4* expression and gene expression in the focal adhesion pathway was also investigated.

### 2.4. Statistical analysis

In this study, R software (v.3.6.2) was used for statistical analysis of all data. The differential expression of *TNS4* between tumor and normal samples was determined with the Wilcoxon test. The median *TNS4* expression level was set as the cutoff value. The OS and progression-free survival (PFS) curves for the high and low *TNS4* expression groups were established with Kaplan–Meier survival analysis. The degree of the impact of *TNS4* expression on survival and other clinical characteristics (gender, age, history of smoking, stage, tumor size, and lymph node status) was determined with univariate and multivariate Cox analyses. The relationship between the clinical pathologic features of the patient and *TNS4* expression was assessed using the Wilcoxon test (for two groups) or the Kruskal–Wallis test (for more than two groups) and logistic regression. *P* values < .05 on both sides were considered statistically significant.

## 3. Results

### 3.1. OS and PFS prognostic analysis of TCGA-LUAD data

**Figure 1.**
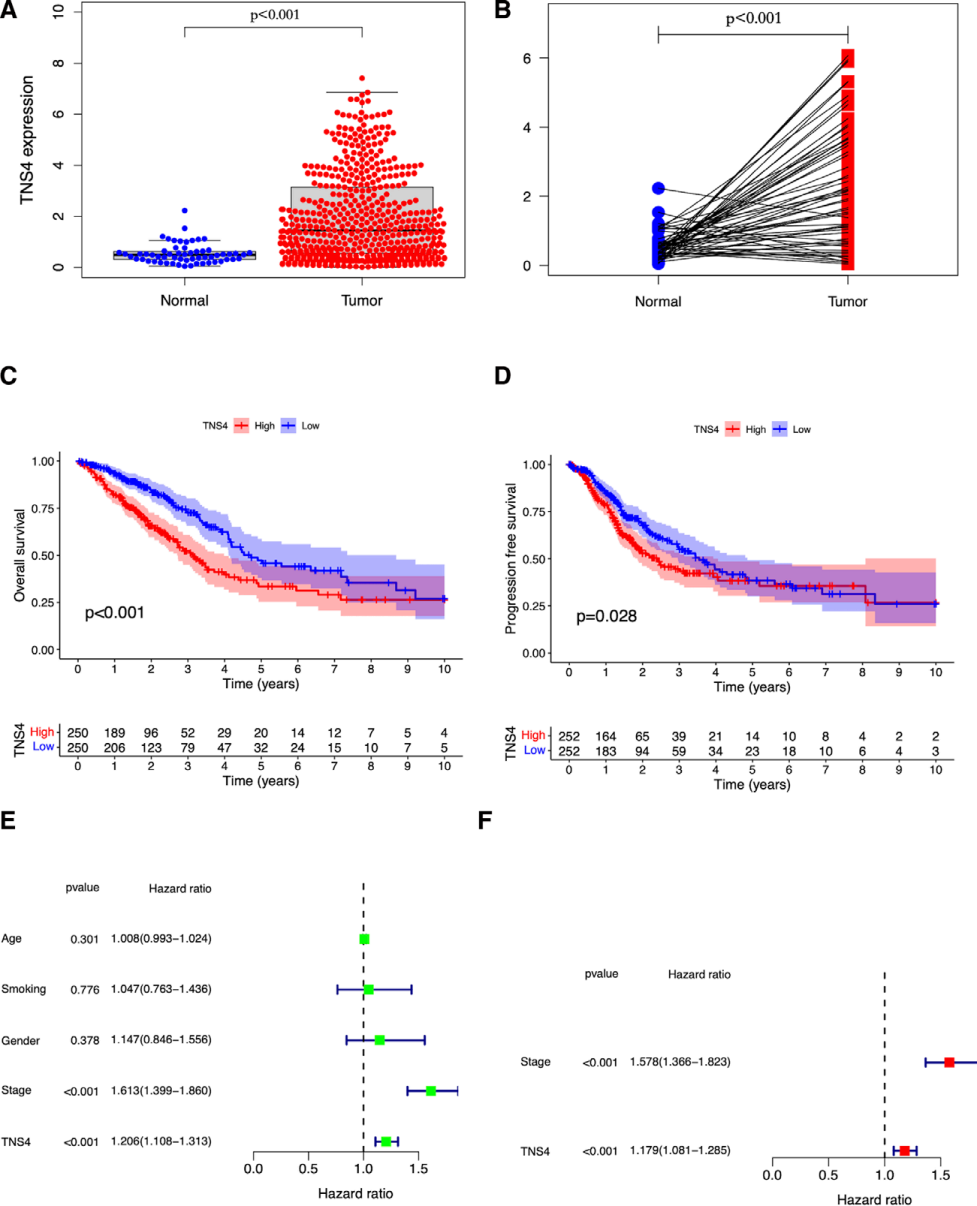
The TNS4 expression in LUAD and its prognostic role in LUAD. The TNS4 is more expressed in tumor tissue than normal tissue by unpaired (a) or paired (b) comparison. LUAD with TNS4 over-expressed has poorer OS (c) and PFS (d). TNS4 expression has a positive correlation with poor overall survival (OS) by both univariate (e) and multivariate analysis (f). PFS = progression-free survival.

TCGA-LUAD contains RNA sequencing data for 535 tumor tissues (including two recurrent tumor samples) and 59 normal tissues collected from 513 patients up to March 2021. After merging the clinical data, 500 cases in the TCGA-LUAD database with RNA sequencing, OS, and clinical data were used for OS prognostic and relationship analyses (Table 1). Five hundred and four cases with PFS data were used for PFS prognostic analysis. The level of *TNS4* expression in all tumor tissues was higher than that in normal tissues (Fig. 1a,b). According to the Kaplan–Meier survival curves, the OS and PFS of LUAD patients with over-expressed *TNS4* were worse than those in LUAD patients with low *TNS4* expression (Fig. 1c,d, *P* < .05). Data for 469 LUAD samples with *TNS4* expression information across all patient characteristics were used to predict survival-time (time-to-event) outcomes with one (univariate) or more (multivariate) predictors using Cox’s proportional hazards model. As shown in Figure 1e, univariate Cox regression analysis indicated that *TNS4* overexpression was significantly correlated with poor OS (HR was 1.21 and 95%CI was 1.11–1.31; *P* < .05). In addition, *TNS4* expression was independently associated with OS in multivariate Cox analysis (HR was 1.18 and 95%CI was 1.08–1.28, *P* < .05).

**Table 1:** TCGA lung adenocarcinoma patient characteristics.

Clinical characteristics	Average (range) or N (%)
Age (yr)	65.3 (33–88)
Gender	500
Male	230 (46.0%)
Female	270 (54.0%)
History of smoking	486
Smoker	415 (85.4%)
nonsmoker	71 (14.6%)
Stage	492
Stage I	268 (54.5%)
Stage II	119 (24.2%)
Stage III	80 (16.3%)
Stage IV	25 (5.1%)
T factor	497
T1	167 (33.6%)
T2	268 (53.9%)
T3	45 (9.1%)
T4	18 (3.6%)
Lymph node status	489
N0	324 (66.3%)
N1	94 (19.2%)
N2	69 (14.1%)
N3	2 (0.4%)

### 3.2. Correlation of *TNS4* expression with clinical characteristics in LUAD patients

**Figure 2.**
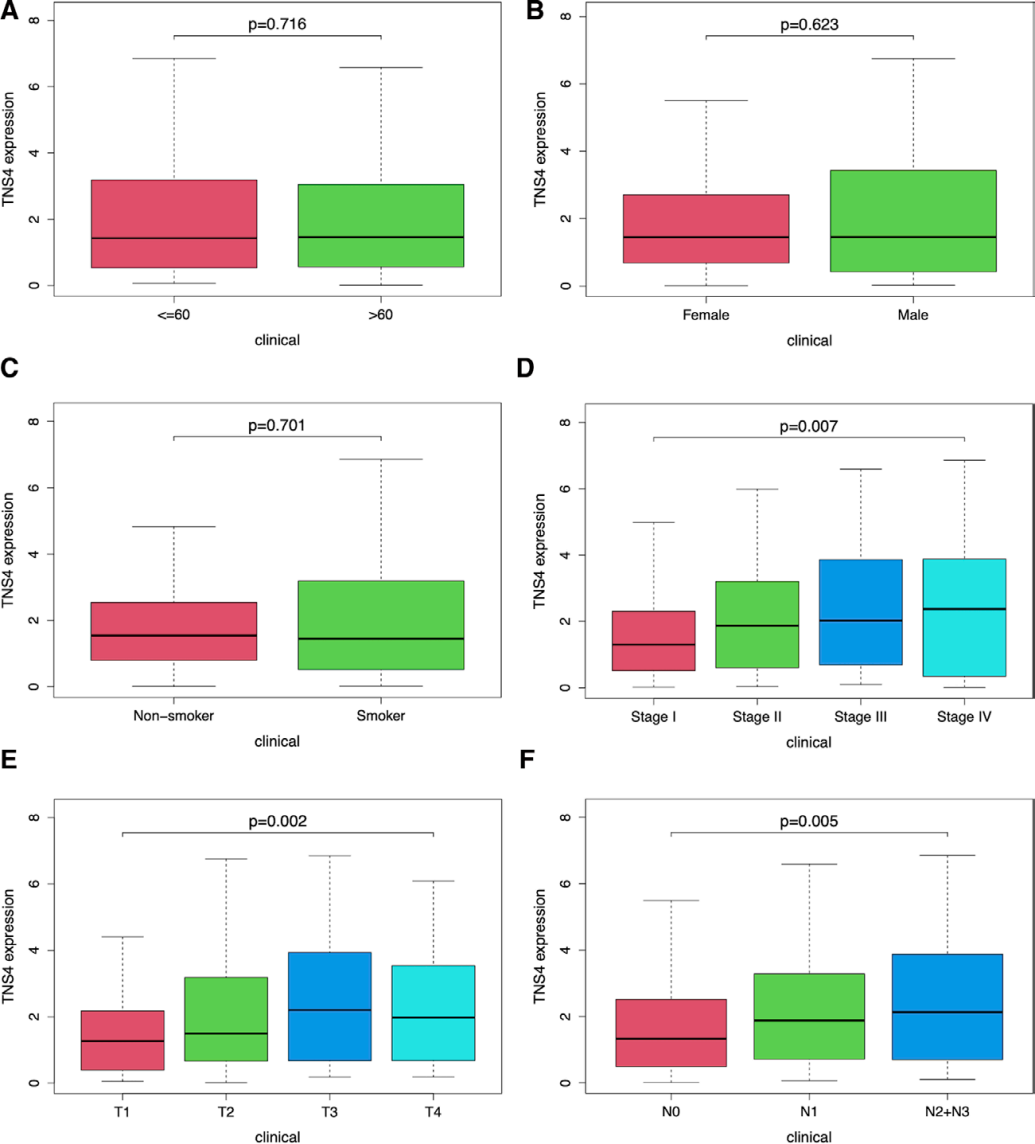
The relationship between TNS4 expression and clinical characteristics including age (a), gender (b), smoking history (c), stage (d), tumor size (e), and lymph nodes involvement (f).

Although *TNS4* expression had no correlation with age, gender, and smoking history (Fig. 2a–c), however, data analysis showed that up-regulated *TNS4* expression was significantly correlated with the LUAD stage (Fig. 2d, *P* < .05), with supporting evidence, such as the T factor (Fig. 2e, *P* < .05) and lymph node status (Fig. 2f, *P* < .05). Logistic regression analysis indicated that patients with high *TNS4* expression had poor prognostic clinicopathologic characteristics compared to those with low *TNS4* expression. Higher expression of *TNS4* was significantly associated with the LUAD status, such as the disease stage (odds ratio [OR] = 1.67 and 95%CI 1.08–2.61, *P* < .05), T factor (OR = 1.56 and 95% CI 1.08–2.28 *P* < .05), and lymph node status (OR = 1.78 and 95% CI 1.22–2.61, *P* < .05) (Table 2).

**Table 2:** TNS4 expression associated with clinical pathological characteristics (logistic regression).

Clinical characteristics	Total number	Odds ratio in TNS4 expression (95% CI)	*p* value
Age (young vs old)	490 (157 vs 333)	1.06 (0.72–1.55)	.771
Gender (female vs male)	500 (270 vs 230)	1.00 (0.70–1.42)	1
Smoking (nonsmoker vs smoker)	486 (71 vs 415)	0.97 (0.58–1.60)	.898
Stage (I + II vs III + IV)	492 (387 vs 105)	1.67 (1.08–2.61)	.022
T factor (T1 vs T2 + T3 + T4)	497 (167 vs 330)	1.56 (1.08–2.28)	.02
Lymph nodes (negative vs positive)	489 (324 vs 165)	1.78 (1.22–2.61)	.003

### 3.3. Meta-analysis for the predictive value of *TNS4* expression in LUAD

**Figure 3.**
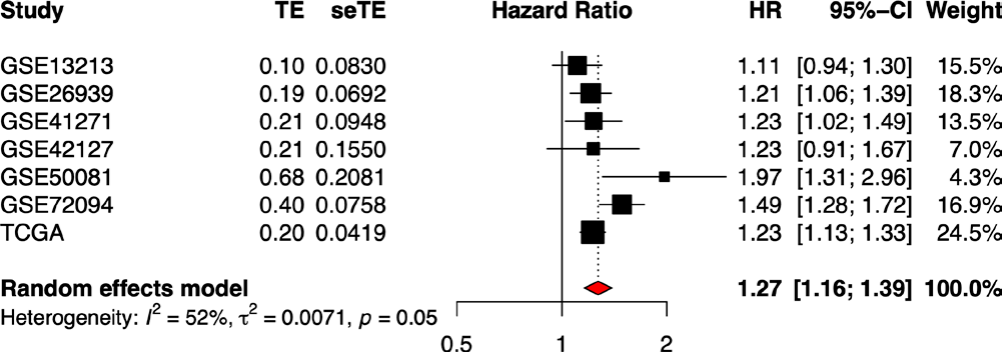
Meta-analysis of LUAD prognosis between high- and low- expression groups of TNS4.

Data obtained from six different datasets were included in our meta-analysis because of the lack of reports on the association between *TNS4* expression and OS among LUAD patients. In the meta-analysis to determine the association between high *TNS4* expression and OS in 1445 LUAD cases, the pooled HR was 1.24 and the 95%CI was 1.18 to 1.31. Furthermore, the heterogeneity among the four datasets was not significant (I2 = 36%, *P* = .17, Fig. 3). All of the results indicated that up-regulated expression of *TNS4* might be considered an important predictor for the OS of LUAD patients.

### 3.4. Identification of *TNS4*-related signaling pathways

**Figure 4.**
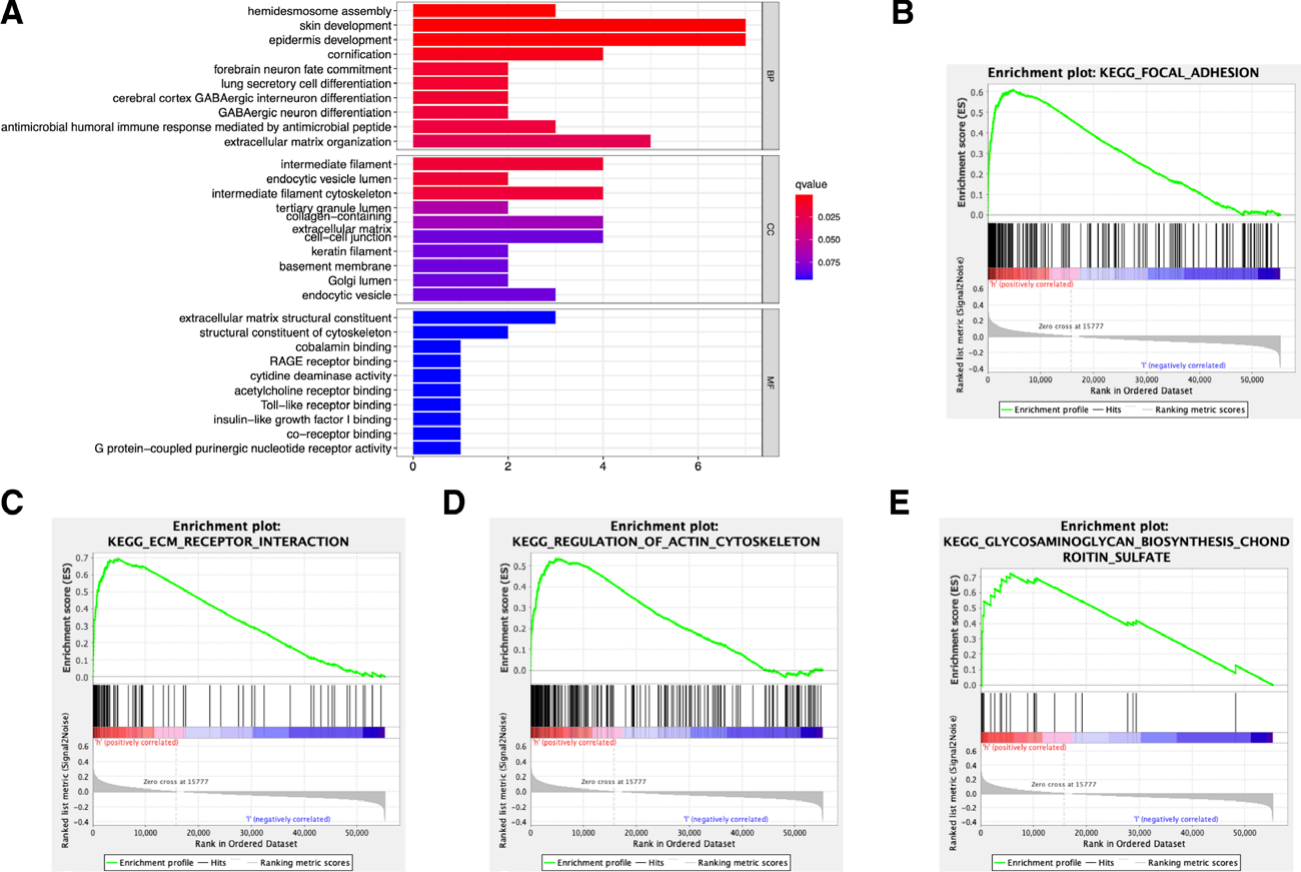
Enrichment analysis of differentially expressed genes between high- and low- expression groups of TNS4. (a) GO analysis. (b) GSEA for KEGG analysis.

**Figure 5.**
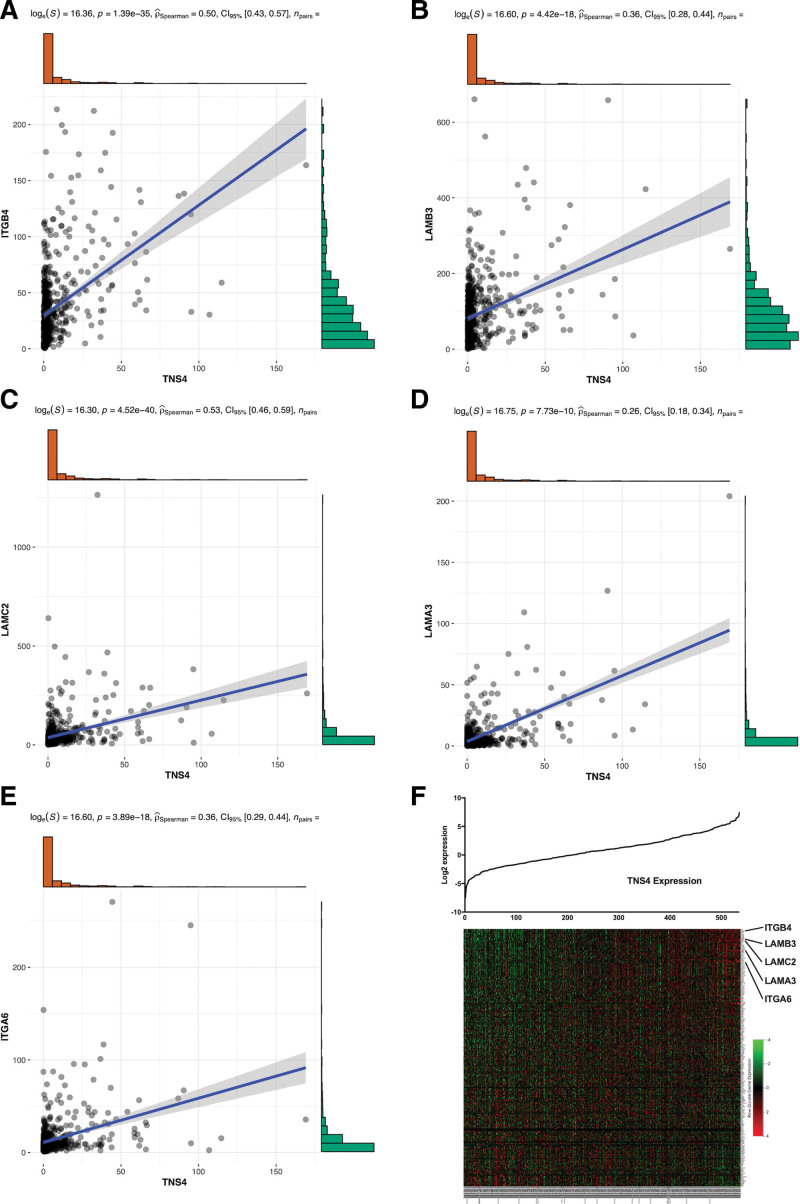
The correlation between expression of TNS4 and other genes in focal adhesion pathway. (a–e) TNS4 has a positive relation with genes coding integrin α6β4 and its ligand, laminin-5. (f) The correlation between expression of TNS4 and genes in focal adhesion pathway. The genes coding integrin α6β4 and laminin-5 have been marked.

In this study, GO and KEGG analyses were used to identify the signaling pathways that are differentially activated between the LUAD patients with high and low *TNS4* expression. GO analysis revealed that some biological processes, cellular components, and molecular functions of the cytoskeleton and extracellular matrix (ECM) were enriched (Fig. 4a). GSEA analysis showed significant differences between high and low *TNS4* expression (FDR < 0.05) in a dataset for the enrichment of molecular signatures (MSigDB Collection; c2.cp: KEGG. V7.1. symbols). In GSEA analysis, the most significantly enriched signaling pathways, based on the normalized enrichment score, were selected including focal adhesion, ECM receptor interaction, regulation of actin cytoskeleton, and glycosaminoglycan biosynthesis - chondroitin sulfate. The first four pathways in the results contained the top four gene sets. The focal adhesion genes were presented as an expression heatmap in Figure 4b–e. Correlation analysis showed that the genes that encode integrin *α*6*β*4 (*ITGB4* and *ITGA6*) and its ligand, laminin-5 (*LAMA3*, *LAMB3*, and *LAMC2*), had a positive correlation with *TNS4* (Fig. 5a–f).

## 4. Discussion and Conclusions

The family of tensin genes has four members, that is, tensin −1, −2, −3, and −4. *TNS4*, located in chromosome 17q12-q21, encodes a protein with 715 amino acids and has been identified as one of the tensin focal adhesion family members. It was recently reported that *TNS4* expression is remarkably up-regulated in human cancers.^[21–23]^ *TNS4* mediates promoter activity in non-small cell lung cancer by inducing epithelial-mesenchymal transition and contributes to the development of resistance to tyrosine kinase inhibitors.^[24]^ To our knowledge, the mechanism underlying the effect of *TNS4* on the prognosis of LUAD has not yet been fully explored. Our bioinformatics study presented in this manuscript provides a more comprehensive prognostic analysis of LUAD. Further, the enrichment analysis results provide possible research directions for the study of the mechanism in the future.

To better understand the prognostic value of *TNS4* expression in LUAD, we performed bioinformatics analysis in this study using high throughput RNA-sequencing data from the public TCGA database. The results showed a close correlation between the increased expression of *TNS4* in LUAD and worse clinical pathologic characteristics, such as high clinical stage, increased tumor size, and lymph node involvement, as well as poor OS and PFS prognosis. Meta-analysis of 6 datasets also confirmed that higher *TNS4* expression could be used as a biomarker for predicting poorer prognosis. Similarly, previous studies have shown that *TNS4* plays a role in promoting tumor invasion and metastasis, implying a correlation between *TNS4* expression and the disease stage.^[25–27]^

To investigate the potential mechanism underlying the role of *TNS4* in the prognosis of LUAD, enrichment analysis was conducted using the GO and KEGG pathways. The results of the functional analysis showed that several pathways were enriched in the high-*TNS4* population, including the cell junction, cytoskeleton, and ECM, which are involved in hemi desmosome assembly, ECM organization, and intermediate filament cytoskeleton in association with the development of many cancers.^[28,29]^ GSEA also showed that several biological processes, such as glycosaminoglycan biosynthesis - chondroitin sulfate, regulation of the actin cytoskeleton, ECM receptor interactions, and focal adhesion were enriched in the high-*TNS4* population. Unsurprisingly, the function of *TNS4* is closely associated with the connection of the ECM to the cytoskeletal networks due to its role in focal adhesions.^[6]^ *TNS4* function is mainly mediated by integrin receptors and their associated protein complexes. In response to extracellular and intracellular stimuli, focal adhesions participate in the regulation of both extracellular and intracellular signaling pathways that regulate cellular events, such as gene expression, proliferation, differentiation, apoptosis, and cell attachment and migration.^[30,31]^ In addition, *TNS4* is known to interact with integrins to promote cell adhesion via stabilizing the *β*1 and *β*4 integrin.^[32]^ The results also indicated that the expression of *ITGA6* and *ITGB4*, the subunits of integrin *α*6*β*4, was positively correlated with the expression of *TNS4*. This may indicate a structural or functional correlation between *TNS4* and integrin *α*6*β*4.

There are several limitations to this study. The present study is a retrospective study based on bioinformatics, and prospective and experimental studies are needed to validate the results obtained. The relation between *TNS4* and integrin *α*6*β*4 is theoretical and should be interpreted with caution. LUAD is a multi-gene-driven disease. In this study, we only focused on *TNS4* expression. However, we cannot conclude that *TNS4* has a direct effect on LUAD prognosis or that it indirectly affects prognosis through other driving genes.

The overexpression of *TNS4* may be one event that occurs during the period of lung cancer development, but the mechanism and relationship with lung cancer remain unclear. We believe that with further in-depth research on the *TNS4* gene, the mechanism for its related genes and pathways in the occurrence and prognosis of lung cancer will be discovered.

In summary, we found that *TNS4* is overexpressed in LUAD and can be used as a prognostic factor for the disease. The molecular mechanisms underlying the clinical value of *TNS4* for predicting lung cancer prognosis may involve structural or functional correlations between *TNS4* and integrin *α*6*β*4, which might be a topic for further investigation. Therefore, targeting *TNS4* might be a potential therapeutic approach for human lung cancer.

## Acknowledgements

We thank Medjaden Inc. for scientific editing of this manuscript.

## Author contributions

**Conceptualization:** Feng Liu, Xinliang Gao, Wujun Xue.

**Data curation:** Feng Liu, Wei Liu.

**Formal analysis:** Feng Liu, Xinliang Gao, Wei Liu.

**Investigation:** Xinliang Gao.

**Methodology:** Xinliang Gao, Wujun Xue.

**Project administration:** Wujun Xue.

**Software:** Xinliang Gao.

**Writing – original draft:** Feng Liu, Wujun Xue.

**Writing – review & editing:** Feng Liu, Wei Liu, Wujun Xue.
